# Predicting Cortical Bone Strength from DXA and Dental Cone-Beam CT

**DOI:** 10.1371/journal.pone.0050008

**Published:** 2012-11-30

**Authors:** Jui-Ting Hsu, Ying-Ju Chen, Ming-Tzu Tsai, Howard Haw-Chang Lan, Fu-Chou Cheng, Michael Y. C. Chen, Shun-Ping Wang

**Affiliations:** 1 School of Dentistry, College of Medicine, China Medical University, Taichung, Taiwan; 2 Stem Cell Center, Department of Medical Research, Taichung Veterans General Hospital, Taichung, Taiwan; 3 Department of Biomedical Engineering, Hungkuang University, Taichung, Taiwan; 4 Department of Radiology, Taichung Veterans General Hospital, Taichung, Taiwan; 5 School of Radiological Technology, Central Taiwan University of Science and Technology, Taichung, Taiwan; 6 Department of Orthopaedics, Taichung Veterans General Hospital, Taichung, Taiwan; Harvard Medical School, United States of America

## Abstract

**Objective:**

This study compared the capabilities of dual-energy X-ray absorptiometry (DXA) and dental cone-beam computed tomography (CBCT) for predicting the cortical bone strength of rat femurs and tibias.

**Materials and Methods:**

Specimens of femurs and tibias obtained from 14 rats were first scanned with DXA to obtain the areal bone mineral density (BMD) of the midshaft cortical portion of the bones. The bones were then scanned using dental CBCT to measure the volumetric cortical bone mineral density (vCtBMD) and the cross-sectional moment of inertia (CSMI) for calculating the bone strength index (BSI). A three-point bending test was conducted to measure the fracture load of each femur and tibia. Bivariate linear Pearson analysis was used to calculate the correlation coefficients (*r* values) among the CBCT measurements, DXA measurements, and three-point bending parameters.

**Results:**

The correlation coefficients for the associations of the fracture load with areal BMD (measured using DXA), vCtBMD (measured using CBCT), CSMI (measured using CBCT), and BSI were 0.585 (*p* = 0.028) and 0.532 (*p* = 0.050) (for the femur and tibia, respectively), 0.638 (*p* = 0.014) and 0.762 (*p* = 0.002), 0.778 (*p* = 0.001) and 0.792 (*p*<0.001), and 0.822 (*p*<0.001) and 0.842 (*p*<0.001), respectively.

**Conclusions:**

CBCT was found to be superior to DXA for predicting cortical bone fracture loads in rat femurs and tibias. The BSI, which is a combined index of densitometric and geometric parameters, was especially useful. Further clinical studies are needed to validate the predictive value of BSI obtained from CBCT and should include testing on human cadaver specimens.

## Introduction

The ability to measure bone strength using noninvasive methods is important for evaluating fracture risk according to the severity of osteoporosis [1], [2], as well as to the early-stage stabilization of artificial implants after implantation in bone (e.g., dental or orthopedic implants) [3], [4]. Dual-energy X-ray absorptiometry (DXA) is one of the methods commonly used in the clinical field of orthopedics for evaluating bone mineral content (BMC) and bone mineral density (BMD) [5]. The areal BMD (in g/cm^2^) measured through DXA is calculated by dividing the obtained BMC (in g) by the projected bone area (in cm^2^) [6]–[8]. Bone quality cannot be determined simply using BMD since, in addition to the intrinsic mechanical quality of the bone, geometric characteristics (size, shape, and macroarchitecture) are important attributes influencing the strength of particular bones [9]. For example, BMD obtained using DXA represents two-dimensional (2D) bone-density information that does not provide data regarding the structural stiffness characteristics, which are related to the bone's shape [6], [10].

Numerous researchers have recently used microcomputed tomography (micro-CT) to measure bone geometry and strength [11]–[13]. Micro-CT also provides three-dimensional (3D) images of bone architecture and various parameters that influence the strength of trabecular bone (e.g., trabecular bone volume, trabecular number, trabecular separation, trabecular thickness, and structure model index). However, because of the limited scan range, micro-CT is mostly used for the bones of small animals (rats or mice) or extracted human bones [12], [14], [15], and is applied more in laboratory-based research than in the clinical field of orthopedics. In clinical orthopedic practice, quantitative computed tomography (QCT) [5], [16], [17] or peripheral quantitative computed tomography (pQCT) [6], [9], [10], [18]–[20] are commonly used to assist in determining the bone BMD. In addition to measuring BMD, pQCT can obtain the 3D geometric parameters of bones. Ferretti and colleagues [7], [9] proposed predicting bone bending strength by using pQCT to measure several bone parameters, including the bone strength index (BSI), cross-sectional moment of inertia (CSMI), and volumetric cortical bone mineral density (vCtBMD). Siu et al. [6] and Moisio et al. [10] suggested that bone strength can be predicted more accurately using pQCT than DXA.

In addition to QCT and pQCT, which are commonly used in orthopedics, in recent years dental computed tomography (CT)—also known as dental cone-beam CT (CBCT)—has been used in the dental field. The resolution of CBCT (typically 75–400 µm) is better than that of traditional CT. Nomura et al. [21] found a linear correlation between the voxel values of CBCT and the contents of hydroxyapatite (HA) rod samples, and some researchers have used CBCT to examine the alveolar bone density of patients to serve as references in presurgical evaluations for dental implants [22]–[24]. Furthermore, the dosage required for CBCT is much less than that for traditional CT [25]–[28], making CBCT an appropriate method for postsurgical follow-up assessments of changes in bone quality [29], [30].

While several studies have evaluated the feasibility of pQCT and DXA for measuring bone strength, few have examined the utility of dental CBCT in assessing cortical bone strength. Most studies have focused on CBCT as a tool for evaluating alveolar bone density before performing dental implantation. Therefore, the present study compared the abilities of DXA and CBCT to predict cortical bone strength in rat femurs and tibias. The hypothesis tested was that the capabilities of CBCT in elucidating and judging BMD and bone shape would make CBCT a better predictor of cortical bone strength than is DXA.

## Materials and Methods

### Specimen Preparation

Fourteen femurs and 14 tibias were collected from 14 healthy male Sprague-Dawley rats [age = 4 months of age, weight = 335±10 g (mean±standard deviation)]. The femurs and tibias of each rat were harvested within 5 min after death. The bone specimens were wrapped with gauze soaked in saline and stored in a −20°C freezer. The study procedures were carried out in strict accordance with the recommendations in the Guide for the Care and Use of Laboratory Animals of the National Institutes of Health. Animal Research Ethics approval was obtained from the Research Ethics Committee of the Taichung Veterans General Hospital (Permit Number: La-101955). All surgery was performed under sodium pentobarbital anesthesia, and all efforts were made to minimize suffering.

### DXA and Dental CBCT Measurements

**Figure 1:**
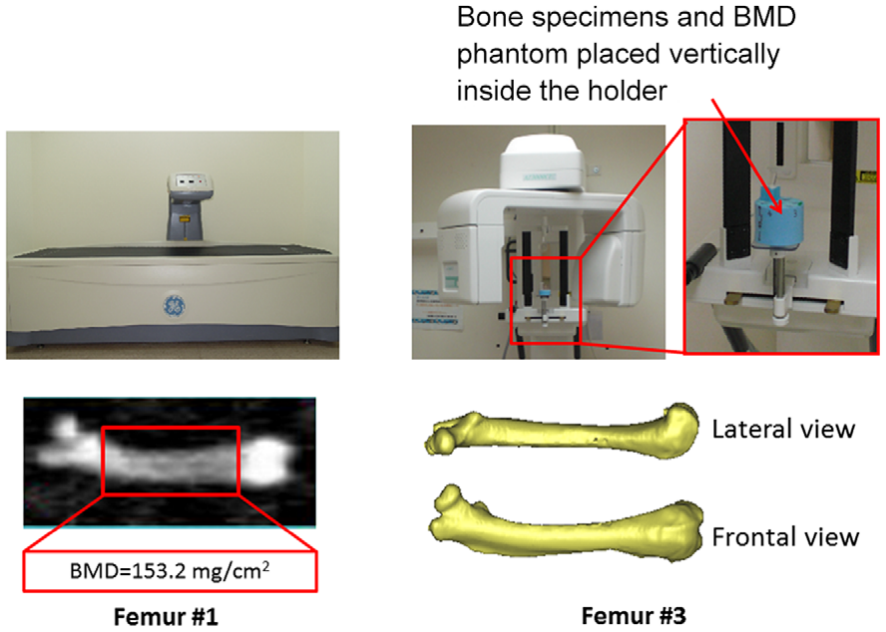
Radiologic measurements of bone specimens: DXA (left) and CBCT (right).

The BMDs of the femur and tibia were measured at the midshaft region using the small-laboratory-animal scan mode of the Lunar Prodigy Advanced System (GE, Madison, WI, USA) (Figure 1). The midshaft regions of the femur and tibia were defined as shown in Figure 1. The BMD was calculated from the BMC of the measured area. The measurement values were calculated automatically using Encore 2007 small-animal software (version 11.20.068, GE, Madison, WI, USA). The DXA machine was calibrated according to the manufacturer's instructions.

**Figure 2:**
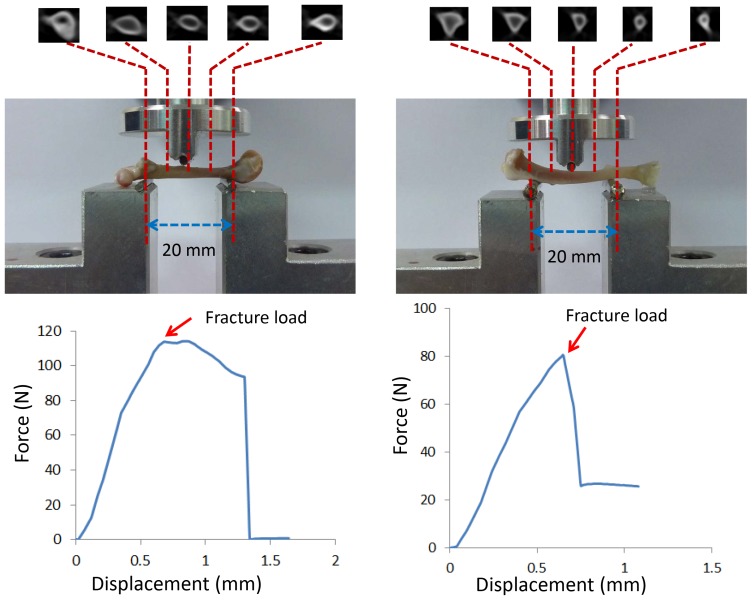
Five CBCT cross-sectional scans, the sites of the DXA scanning measurements, and the three-point bending test: femur (left) and tibia (right). (The force-vs-displacement curves were recorded using the three-point bending test for femur #6 and tibia #10.)

A dental CBCT device (AZ 3000, Asahi Roentgen, Japan) was used to obtain the CBCT images of each femur and tibia (Figure 1). The scanning parameters were set at 85 kV, 3 mA, and a voxel resolution of 155 µm. When performing the CBCT scans for all bone specimens, two phantoms with predetermined HA concentrations [0.25 and 0.75 g/cm^3^ HA BMD phantoms obtained from Skyscan (Skyscan, Aartselaar, Belgium)] were constructed in order to calculate the vCtBMD of the bones. The obtained CBCT images were loaded into professional medical imaging software (Mimics, Materialise, Leuven, Belgium) to calculate the vCtBMD values (in g/cm^3^) of the midshaft portions of the femurs and tibias (Figure 2). Five images of the midshaft portion of each femur and tibia were then imported into ImageJ 1.45 s (Rasband, W.S., ImageJ, US National Institutes of Health, Bethesda, Maryland, USA) [31] to measure the CSMI (in mm^4^) of the femurs and tibias and for finally calculating the BSI ( = vCtBMD×CSMI) [9].

### Three-Point Bending Test

After removing their soft tissues, each femur or tibia was placed on a specially designed loading apparatus on a material testing system (JSV-H1000, Japan Instrumentation System, Nara, Japan), as shown in Figure 2. Loads were applied at distances of 40% and 45% of the total femoral and tibial lengths from the anatomic inferior side. The two supporting locations were separated by 20 mm (Figure 2). A static preload of 1 N was applied to fix the bone specimens between the contacts. The loading speed of the crosshead was set to 20 mm/min using the displacement control mode. The force-vs-displacement data were acquired and recorded at a sampling rate of 40 points/second until the bone specimen was fractured. The strength (fracture load) was determined as the highest point of the obtained curve.

### Statistical Analysis

The mean, standard deviation, and coefficient of variation (CV) were calculated for all measurements. The Shapiro-Wilk test was used to determine if the measurements conformed to a normal distribution. The paired-sample *t*-test was used to compare differences between the measurements and the results of the CBCT, DXA, and three-point bending test between the femurs and tibias from the same rat. A bivariate linear Pearson analysis was used to calculate the corresponding correlation coefficients (*r* values). All statistical analyses of the data were performed using OriginPro software (Version 8, OriginLab, Northampton, MA, USA). The level of the statistical significance was set at *P*<0.05.

## Results

### Densitometric, geometric, and mechanical test results

The measured densitometric, geometric, and mechanical parameters of the rat femurs and tibias are summarized in Table 1. All of the experimental data were normally distributed (*p*<0.05). The CV was largest for the BSI (32.75% and 35.11% for femurs and tibias, respectively) and smallest for the vCtBMD (6.74% and 8.06%). The densitometric parameters (BMD measured by DXA and vCtBMD measured by CBCT), geometric parameter (CSMI measured by CBCT), combined densitometric and geometric parameters (BSI, = CSMI×vCtBMD), and fracture load were all significantly higher for the femurs than for the tibias (*P*<0.001).

**Table 1:** Experimentally measured densitometric and geometric parameters of the femurs and tibias obtained from DXA and CBCT. The fracture loads based on the three-point bending test are also listed.

	Parameter	Unit	Femur (N = 14)		Tibia (N = 14)		P values
			Mean±SD	CV(%)	Mean±SD	CV(%)	
DXA	BMD	mg/cm^2^	133.69±13.69	10.23	103.29±10.34	10.00	<0.0001
CBCT	vCtBMD	mg/cm^3^	1264.48±85.21	6.74	936.50±75.44	8.06	<0.0001
	CSMI	mm^4^	8.58±2.55	29.68	3.98±1.17	29.36	<0.0001
	BSI		10937.58±3582.43	32.75	3775.20±1325.60	35.11	<0.0001
MT	Fracture load	N	111.26±12.63	11.25	98.51±13.93	14.14	0.006

SD = standard deviation; CV = coefficient of variation (SD/mean×100%); DXA = dual-energy X-ray absorptiometry (DXA); CBCT = cone beam computed tomography; MT = mechanical test (three-point bending test); BMD = bone mineral density; vCtBMD = volumetric cortical bone mineral density; CSMI = cross-sectional moment of inertia; BSI = bone strength index.

### Correlations between radiologic measurements and mechanical test results

**Figure 3:**
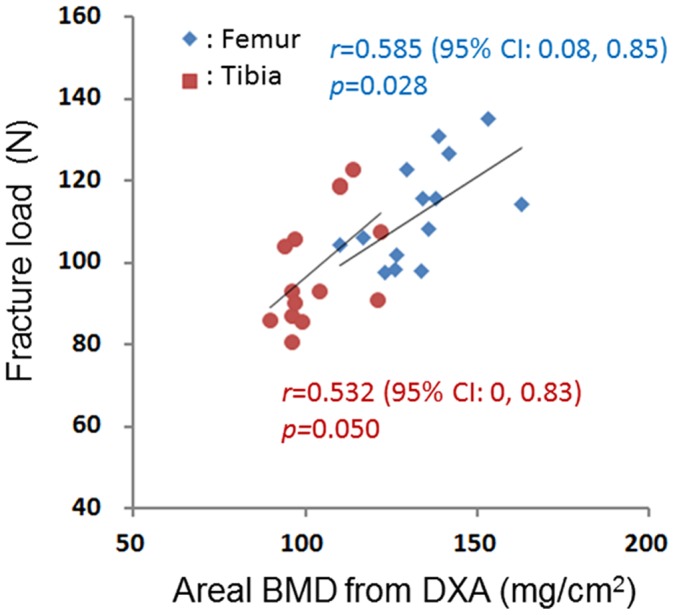
Correlation between fracture load (measured using a three-point bending test) and areal BMD (measured using DXA).

**Figure 4:**
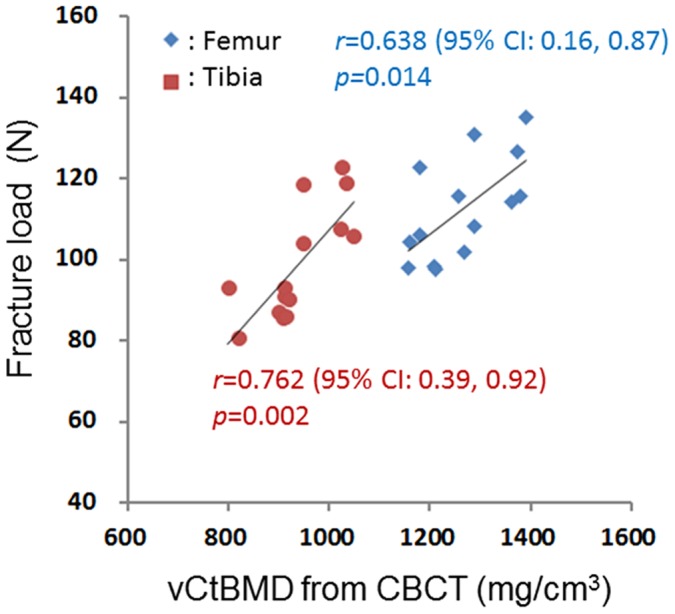
Correlation between fracture load (measured using a three-point bending test) and vCtBMD (measured using CBCT).

**Figure 5:**
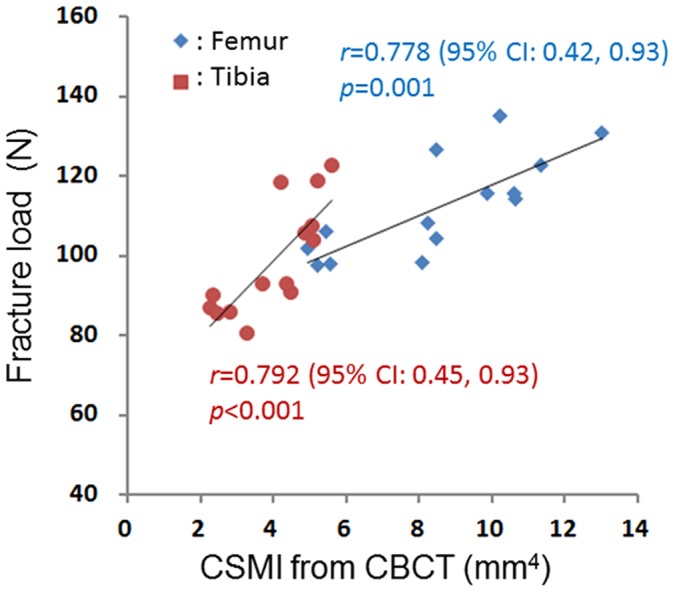
Correlation between fracture load (measured using a three-point bending test) and CSMI (measured using CBCT).

**Figure 6:**
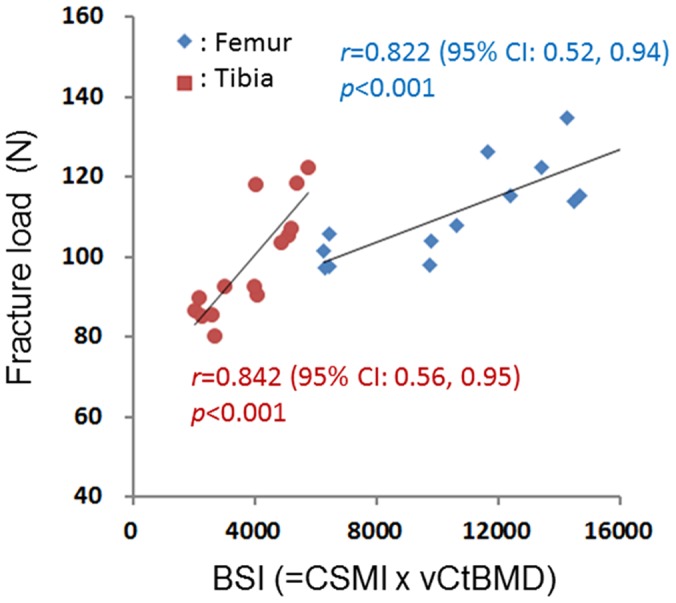
Correlation between fracture load (measured using a three-point bending test) and BSI ( = CSMI×vCtBMD).

The correlation coefficients for the associations of the fracture loads with the areal BMD (measured using DXA), vCtBMD (measured using CBCT), CSMI (measured using CBCT), and BSI (combined densitometric and geometric parameters) were 0.585 (*p* = 0.028) and 0.532 (*p* = 0.050) (for the femur and tibia, respectively; Figure 3), 0.638 (*p* = 0.014) and 0.762 (*p* = 0.002) (Figure 4), 0.778 (*p* = 0.001) and 0.792 (*p*<0.001) (Figure 5), and 0.822 (*p*<0.001) and 0.842 (*p*<0.001) (Figure 6), respectively. The strong associations meant that the following linear expressions accurately describe the fracture load for the bone specimens: Fracture load_Femur_ = 0.0029 BSI_Femur_+80.562 and Fracture load_Tibia_ = 0.0088 BSI_Tibia_+65.123.

## Discussion

It would be very helpful if noninvasive methods could be used to accurately measure the BMD in order to predict bone strength. DXA has been the most common method, but it only provides 2D information regarding bone density and hence is of limited use in determining the strength of an actual bone structure. Previous studies have shown that compared to DXA, pQCT provides more information about the geometric parameters of bones, and is superior for predicting bone strength [6], [10]. Dental CBCT has recently become a popular method for evaluating alveolar bone density prior to dental implantation [22]–[24]. Researchers have recognized the ability of CBCT to predict BMD [21], although no studies have predicted the strength of long bones using dental CBCT. The present study is the first to evaluate the bone strength of cortical bones using dental CBCT, and the obtained results indicate that CBCT is superior to DXA for predicting cortical bone fracture loads.

Bone strength is commonly used in evaluations of the risk of bone fractures. Both the material (or densitometric) parameters (i.e., the intrinsic mechanical quality of the bone) and the geometric parameters of the bone shape (i.e., the size, shape, and macroarchitecture) affect bone strength. Therefore, the 2D areal BMD (measured using DXA) cannot be used to accurately determine the bone strength [9]. The correlation coefficient between the areal BMD of rat femurs as measured using DXA and fracture loading was found to be 0.585 (*p* = 0.028). This represents a moderate correlation, slightly weaker than that obtained by Siu et al. [6] (*r* = 0.612) for the correlation between fracture load and areal BMD (measured using DXA) for the femurs of 23 goats. These different findings can be attributed to the sample variance of the two studies. The variances among the rats included in the present study (age = 4 months, weight = 335±10 g) were small, leading to a narrower distribution of the BMD for the femurs with a CV of 10.23%, which was smaller than the value of 15.0% found by Siu et al. (age = 4–6 years, weight = 26–30 kg).

The measurements of the geometric parameters of bones obtained using noninvasive methods could be useful when evaluating bone strength. Although micro-CT provides several parameters for trabecular and cortical bones, it is not commonly used in clinical settings because it can only be applied to small specimens. Siu et al. [6] and Moisio et al. [10] studied goat femurs, goat humeri, and beagle femurs, and found that the BSI of the midshaft cortical bone portions of long bones measured using pQCT yielded better predictions of the bone fracture load than did the areal BMD obtained using DXA. These findings imply that pQCT not only measures densitometric parameters (volumetric BMD) of bones but also—with the addition of the geometric parameters (i.e., CSMI) of bones—can more accurately predict bone strength.

Dental CBCT is characterized by lower cost, smaller spatial volume, and lower radiological dosages relative to traditional CT, which has made CBCT popular in clinical dental diagnosis and treatment services. CBCT can be used to determine the shape of bones precisely [32], [33] due to its ability to differentiate among bone tissues. In addition, its higher spatial resolution means that it should be possible to use it to measure the CSMI of rat femurs and tibias accurately, such as those used in this study. In addition to the ability of CBCT to precisely measure geometric shapes, recent studies have examined its effectiveness in determining the bone BMD. Nackaerts et al. [34] showed that the intensity values in CBCT were not reliable because they are influenced by the actual device used and the sample positioning. However, most CBCT models use a flat panel detector (FPD) instead of image intensifier (I.I) type [21]. The FPD is quiet, has a wider dynamic range than I.I., and provides improved image quality [35]. In addition, Nomura et al. [36] demonstrated that there was strong correlation between the voxel values and concentrations of iodine solutions, but that the variance of the voxel values was larger than the CT number from multiplied CT. Nomura et al. [21] have recently indicated that CBCT might be able to determine BMC from the voxel values of dental CBCT. Therefore, the present study used dental CBCT to measure the vCtBMD of rat bones.

Animal bones are typically used in biomechanical studies since human cadaveric bones are difficult to obtain. Although the results of studies that use bones from large animals with similar bone structures to humans, such as dogs and goats, are more appreciated, many studies have used rat femurs or tibias in such tests [9], [19], [37], [38]. In addition, although the three-point bending test does not measure the pure bending moment, since this type of measurement includes the shear stress, it is used for measuring bone strength [6], [9], [10], [18], [19], [37], [39], [40] more often than are compression, torsion, and tension tests. Furthermore, the groups in this study were larger (comprising 14 samples/group) than the recommended minimum of 11 samples proposed by Leppanen et al. [41] when applying the three-point bending test to evaluate the bone fracture load.

The fracture load was slightly higher for the femurs (111.26±12.63 N) than for the tibias (98.51±13.93 N). This contrasts with Jamsa et al. [18] finding that the fracture load was slightly higher for mouse tibias (21.1±6.4 N) than for mouse femurs (19.9±4.3 N) when using a three-point bending test, which may reflect differences in the bone structure between rats and mice. We further analyzed the findings of studies that used rat femurs. The measured femur fracture load was 111.26±12.63 N in our study, while it was 162.22±17.06 N, 163±26.1 N (for a daily intake of 2000 ppm Mg), and 340±60 N (estimated from figures) in the studies of Jiang et al. [19], Stendig-Lindberg et al. [39], and Iwamoto [42], respectively. In addition to factors such as the type, age, and weight of the rats possibly leading to significantly different results, the span distance between the two ends in the three-point bending test is a primary factor influencing the measured fracture load. In the present study the span distance was set to 20 mm, which was wider than that used in previous experiments and therefore yielded a lower fracture load. However, the rat femur vCtBMD densitometric parameter was slightly smaller in this study (1264.48±85.21 mg/cm^3^) than those obtained by Leppanen et al. [40] (1431±334 mg/cm^3^) and Jiang et al. [19] (1334.15±11.97 mg/cm^3^), while the femur CSMI geometric parameter obtained in this study (8.58±2.55 mm^4^) was slightly larger than that obtained by Leppanen et al. (7.85±2.29 mm^4^).

Our experimental results show that the correlation coefficients of the obtained BSI ( = CSMI×vCtBMD) with the femur and tibia fracture loads were 0.822 and 0.842, respectively. These values are lower than that of 0.94 obtained by Ferretti et al. [9] for between the fracture load and the BSI of rat femurs measured using pQCT. This could be mainly attributable to Ferretti et al. [9] using both rats that had normal bone quality and those that were treated with dexamethasone or aluminum hydroxide, which is likely to have resulted in a larger variation in the cortical BMD values of the specimens. However, Siu et al. [6] found (using pQCT) a correlation coefficient of only 0.334 between BSI_CSMI_ and the fracture load of goat femurs whose quality was similar to that of the normal bone in the present study. Nevertheless, using BSI_cross-sectional area_ as an indicator to predict the fracture load of femurs increased the correlation coefficient to 0.697. In addition, Moisio et al. [10] also used pQCT to measure beagle femurs, and found that the adjusted *r* ^2^ between the BSI and the fracture load was 0.877. Both the CBCT used in the present study and the pQCT used in previous studies to measure the combined densitometric and geometric parameters of bones, such as BSI ( = vCtBMD×CSMI or vCtBMD×cross-sectional area), yield better predictions of bone strength than the areal BMD that is measured using DXA. These results show that, in addition to pQCT, CBCT is an appropriate method for evaluating the strength of cortical bone (quantified as the fracture load).

The limitations of this study should be considered. First, because of the difficulty of obtaining human cadaveric bones, rat bones were used in this preliminary study. Although the results of this study are based on a rat model, the described experimental procedure could be applied to human bones when they become available. Second, only 14 femurs and tibias were used in this study. Although this is more than the 11 samples proposed by Leppanen et al. [41] when applying the bending test to evaluate the bone fracture load, more samples may be needed to better quantify the association between fracture load and measured parameters. Third, this study used dental CBCT to evaluate only the cortical bone strength, and so future studies should explore the ability of CBCT to predict the strength of trabecular bone. Fourth, the bone strength is affected not only by densitometric (density) and geometric (CSMI) parameters, but also by other factors such as inhomogeneity (in the bone density distribution), anisotropy, and the strain rate.

## Conclusions

Based on the results obtained from rat bones *in vitro*, the vCtBMD, CSMI, and BSI obtained using dental CBCT all provided superior predictions of cortical bone bending fracture loads than did areal BMD measured using DXA. Furthermore, strong correlations were found between the BSI ( = vCtBMD×CSMI) and the fracture loads (*r* = 0.822 and 0.842 for femurs and tibias, respectively). Subject to the limitations of the sample size and the experimental setup, dental CBCT is a noninvasive method that requires low radiological dosages to predict bone strength, and might constitute a suitable alternative to pQCT, especially when frequent radiological examinations must be conducted within a short time period.
